# Supplementary figures and images for: Potent kinase inhibitors from the Merck KGaA OGHL: Novel hits against Trypanosoma brucei with potential for repurposing
Source: PLoS Negl Trop Dis. 2025 Nov 11;19(11):e0013719. doi: 10.1371/journal.pntd.0013719 (PMC12622802; doi:10.1371/journal.pntd.0013719)

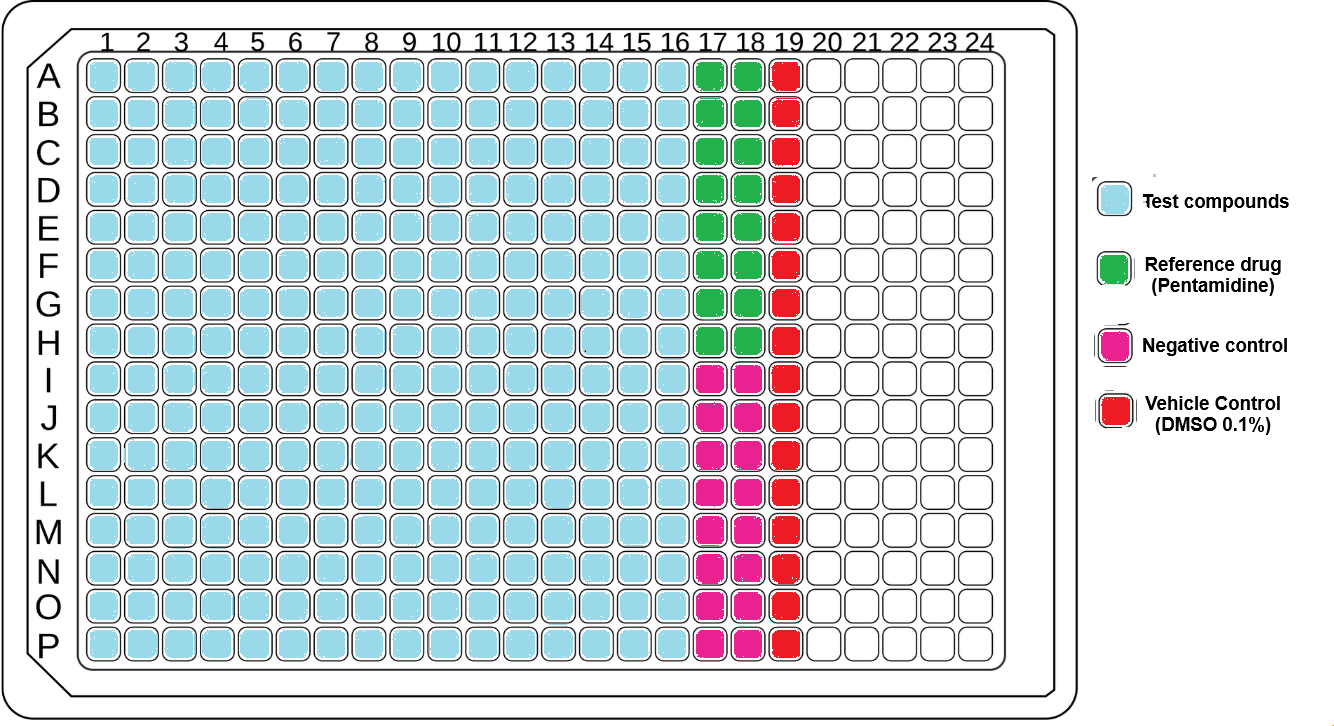

Supplement: S1 Fig — The mapping shows the test compounds panel, positive control (reference drug- pentamidine) panel, negative control (untreated parasites) panel, and vehicle (0.1% DMSO) panel. (TIF) [file pntd.0013719.s001.tif]
