# Supplementary material for: Potent kinase inhibitors from the Merck KGaA OGHL: Novel hits against Trypanosoma brucei with potential for repurposing
Source: PLoS Negl Trop Dis. 2025 Nov 11;19(11):e0013719. doi: 10.1371/journal.pntd.0013719 (PMC12622802; doi:10.1371/journal.pntd.0013719)
Supplement: Attachment — Submitted filename: Response to Reviewers.docx [file pntd.0013719.s003.docx]

**PNTD-D-25-01282
Potent Kinase Inhibitors from the Merck KGaA OGHL: Novel Hits against Trypanosoma brucei with Potential for Repurposing**

**PLOS Neglected Tropical Diseases**

**Response to Reviewers**

**Additional Editor Comments:**

I tend to agree with comments of the reviewer that the study needs clearer methodological details. Specifically, (1) please include a vehicle control to satisfy the author concerns. (2) it will be better to add one plate layout (as supplementary material) of the drugs and control included in the plate and (3) make the acknowledgement more explicit to Merck KGaA, Darmstadt, Germany, and the Open Innovation Portal, preferably in methodology section provide their portal link and how they help the research of new molecules.

Response: Thank you for these valuable suggestions. We have now included the vehicle control (0.1% DMSO), provided a detailed plate layout as Supplementary Figure S1, and expanded the acknowledgement to Merck KGaA, Darmstadt, Germany, highlighting their contribution to compound access in the methodology section.

**Journal Requirements:**

Please upload all main figures as separate Figure files in .tif or .eps format. For more information about how to convert and format your figure files please see our guidelines:

https://journals.plos.org/plosntds/s/figures

Response: Figures are provided as separate files in .tif format.

**Reviewers' Comments:**

Key Review Criteria Required for Acceptance?

**Methods:**

**Reviewer #1:** The methodology section is unclear. It missed important details.

**Line 115**: DMSO has cytotoxic effect on the cells. Justify its use as a solvent in your work on cell cultures. Moreover, it may have an anti-parasitic effect on Trypanosomes. Mention how to exclude this effect in your study.

**Response**: Thank you for raising this important point. All compounds from MERCK were supplied as 10mM DMSO stocks. Upon dilution in incomplete culture media for activity testing, the DMSO concentration was reduced to 0.1% that has no documented effect on trypanosomes and cell viability. Moreover, 0.1%DMSO was included in our experiments as vehicle control. The methodology section was revised accordingly.

**Line 118**: Define T. b. brucei Lister 427 as a virulent lab strain of Trypanosoma brucei

**Response**: Thank you for the suggestion. We have added the description of T. b. brucei Lister 427 as a virulent laboratory strain in the methodology section.

**Line 120**: add the principle of resazurin reduction assay

What are the reference drugs and the negative control?

**Response**: Thank you. The principle of resazurin reduction assay is now included in the “**Potency assessment of compounds against *T. b. brucei*”** section.

Pentamidine isethionate was used as the reference drug (positive control), untreated parasites as the negative control, and 0.1%DMSO as the vehicle control.

**Lines 138 and 139**: for a full dose-response assay, a single concentration of each compound and the positive control is not sufficient. This is not a proper control for a dose-response curve, as a dose-response curve requires multiple concentrations.

**Response**: Thank you for your remark. Please, note that prior to the full dose-response assay (section: Antitrypanosomal dose-response studies of the compounds), we performed a single concentration point screening of compounds at 10 µM to select those qualifying (≥ 90% inhibition) for dose-response studies. We have now provided more clarity in the methodology section.

**Line 139**: The incubation time for the cytotoxicity assay is stated as 42 hours, which is an unusual duration. Most cell viability assays use either 24, 48, or 72 hours. This should be justified.

**Response**: We apologize for the typographic error. We have now changed the incubation time to **48 hours** as appropriate.

**Line 148**: state the actual concentrations used

**Response**: Thank you. We have now clearly stated the actual concentrations used in the experiments.

**Line 154**: Missing reagent in the sentence ”washed three times with…”

**Response**: Thank you for noting this. The missing reagent (phosphate buffer saline (DPBS 1X)) has been added to the sentence.

**Line 155**: "1/100 drug dilution," is contradictory to resuspending in "drug-free medium.

**Response**: Thank you for your remark, "1/100 drug dilution" is now deleted from the sentence.

**Line 150 and 156**: re write the enumeration of motile cells

**Response**: Thank you. The section now read as “To evaluate the killing kinetics of selected compounds, bloodstream forms of *T. b. brucei* (2 x 10⁵ cells/mL) were incubated in normal culture conditions in the presence of compounds at 8IC_50_, 4IC_50_, IC_50_, and 0.25IC_50_ concentrations in 24-well plates. Upon incubation, cultures were centrifuged at 0, 4, 8, 12, 24, 30, 36, 48, 60, and 72 hours’ time intervals at 2,500 rpm followed by motile trypanosomes enumeration using a Lumascope LS520 inverted microscope [17]”

**Line 227**: reevaluation of the powdered forms of some compounds should be added in the methodology.

Why did the authors choose these three compounds only to test for the powdered forms?

**Response**: Thank you for your comment. We have now included a section, namely “**Confirmatory dose-response studies”** to describe the re-evaluation of the powdered forms of selected compounds.

The three compounds were selected for confirmatory studies based on their highest antitrypanosomal activity (IC_50_ ≤1µM) and good selectivity (SI ≥50 µM).

**Line 228**: It is not clear which reagent was used to dissolve them.

**Response**: Thanks for raising this out. DMSO was used to dissolve the powdered compounds. We have now specified this information in the “**Confirmatory dose-response studies”** section.

Reviewer #2: The introduction clearly demonstrates the urgent need for new trypanocidal agents and the study objective is to identify novel hits from the Merck KGaA Open Global Health Library (OGHL) and rationalize their mechanisms

Hypothesis is Implicit but testable.

Study design aligns with the stated objectives, SI values were calculated, and data were presented with mean ± SD.

Since the work used in vitro parasite and mammalian cell cultures, there are no direct human/animal subjects requiring ethics approval.

**Results:**

**Reviewer #1:** The results section is redundant and confusing

It lacks documentation of different assays and cultures used.

What are the effect of the tested compounds on trypanosomes themselves?

**Response**: Thank you for your remarks. We have now revised the Results section to remove redundancy while taking away methodological details. We also specified the different assays and parasite cultures used where necessary. Regarding the effect of the tested compounds on T. brucei, we clarified this in the Results section (lines 341–363). Briefly, compounds OGHL00006 and OGHL00169 exhibited irreversible trypanocidal activity, while compound OGHL00133 showed a reversible inhibitory effect, as parasites were able to recover following drug withdrawal.

**Conclusions:**

**Reviewer #1**: Some conclusions are not supported by the data presented. for example, Line **546**: The authors mentioned that “The ability of these compounds to disrupt conserved pathways” . This conclusion is based on which results???

What are the limitations of the current study?

**Response**: Thank you for the remark. We have now revised the statement “The ability of these compounds to disrupt conserved pathways…” to read “The *in silico* activity of these compounds against conserved targets between parasitic and human cells support the hypothesis that kinase inhibitors may hold potential as antiparasitic agents, although further experimental validation is required”.

We have also included statements on limitations of the study at the end of the discussion section.

**Reviewer #2:** This research paper is highly relevant to public health. The introduction and author summary emphasize that African trypanosomiasis remains a critical health and socio-economic burden, complicated by drug resistance and limited treatment options.

Some limitations are acknowledged, but they need clearer articulation.

I recommend adding a short, explicit “Limitations” paragraph in the Discussion.

**Response**: Thank you for your suggestion. We have now added a limitation statement at the end of the discussion section.

**Summary and General Comments:**

**Reviewer #1:** The authors presented a manuscript regarding the effect of different compounds. However, a detailed methodology and clear results should be presented.

**Response**: Thank you for your remark. As stated above, we have thoroughly revised the manuscript to provide more detailed methodological descriptions and clarity in the results presentation. All the suggestions and comments have been carefully considered and incorporated to improve the overall clarity and quality of the manuscript.

**Reviewer #2**: Overall, this manuscript does address all the checklist points. The only possible gap is the lack of an explicitly worded hypothesis statement and formal statistical power justification. Everything else, objectives, design, population, methods, analyses, and results is clear and appropriate.

**Response**: Thank you for your comment. We have now added a hypothesis statement at the end of introduction. In addition, statistical data analysis is now included at the end of the methodology section.
