# Supplementary material for: Potent kinase inhibitors from the Merck KGaA OGHL: Novel hits against Trypanosoma brucei with potential for repurposing
Source: PLoS Negl Trop Dis. 2025 Nov 11;19(11):e0013719. doi: 10.1371/journal.pntd.0013719 (PMC12622802; doi:10.1371/journal.pntd.0013719)
Supplement: Attachment — Submitted filename: Response_to_Reviewers_auresp_2.docx [file pntd.0013719.s004.docx]

**PNTD-D-25-01282
Potent Kinase Inhibitors from the Merck KGaA OGHL: Novel Hits against *Trypanosoma brucei* with Potential for Repurposing**

**PLOS Neglected Tropical Diseases**

**Response to Reviewers**

**Reviewer #1**

**Methods**

Line 162:  Use the sentence provided in your response [The three compounds were selected for confirmatory studies based on their highest antitrypanosomal activity (IC50 ≤1μM) and good selectivity (SI ≥50 μM)]
instead of
[Confirmatory dose-response assays were conducted for the most active and selective compounds identified during the primary screening].

Response: Thank you for this suggestion. The sentence “Confirmatory dose-response assays were conducted for the most active and selective compounds identified during the primary screening” is now changed to “Three compounds were selected for confirmatory studies based on their highest antitrypanosomal activity (IC_50_ ≤1μM) and good selectivity (SI ≥30).”

Thank you very much for giving us the opportunity to upgrade our manuscript.
